# Supplementary material for: CBM: An IoT Enabled LiDAR Sensor for In-Field Crop Height and Biomass Measurements
Source: Biosensors (Basel). 2021 Dec 29;12(1):16. doi: 10.3390/bios12010016 (PMC8774002; doi:10.3390/bios12010016)
Supplement: Supplementary file 1 [file biosensors-12-00016-s001.zip › biosensors-1497794-supplementary.pdf]

biosensors

MDPI

Article

CBM: An IoT Enabled LiDAR Sensor for In-Field Crop Height and Biomass Measurements

Bikram Pratap Banerjee [1], German Spangenberg [2,3] and Surya Kant [1,2,3,*]

[1] Agriculture Victoria, Grains Innovation Park, Horsham VIC 3400, Australia; bikram.banerjee@agriculture.vic.gov.au
[2] Agriculture Victoria, AgriBio, Centre for AgriBioscience, Bundoora VIC 3083, Australia; german.spangenberg@agriculture.vic.gov.au
[3] School of Applied Systems Biology, La Trobe University, Bundoora VIC 3083, Australia
* Correspondence: surya.kant@agriculture.vic.gov.au; Tel.: +61-3-4344-3179

Table S1. List of primary parameters for the 3D printer.

Sr. No.	Parameters	Values
1	Layer Height	0.1 m
2	Wall thickness	1.2 m
3	Infill Density	100%
4	Infill Pattern	Cubic
5	Printing Temperature	245 ºC
6	Build Plate Temperature	85 ºC
7	Print Speed	25 mm/s
8	Cooling Fan speed	20 %

3D printer (Ultimaker S5, Geldermalsen, The Netherlands).

Table S2. Wheat genotypes used in the field trial.

Sr. No.	Wheat Genotypes	Growth Type
1	Aus27214	Spring
2	Aus428	Spring
3	Aus27919	Spring
4	Aus27319	Spring
5	Aus79	Spring
6	HOPE	Spring
7	Aus27919	Spring
8	7992	Spring
9	31B-55	Spring
10	Aus26558	Spring
11	A4920-52P-2P-2P	Spring
12	Aus28110	Spring
13	Aus27258	Spring
14	178/89	Spring
15	Cara	Spring
16	Aus28232	Spring
17	SKOROSPELKA Aus36	Spring
18	Aus28040	Spring
19	Yitpi	Spring
20	DIAMOND	Spring
21	KLEIN DON ENRIQUE	Spring
22	Emma	Spring
23	Aus27959	Spring
24	Aus27858	Spring
25	Aus17	Spring
26	MAGPIE S	Spring
27	HINDI 12	Spring

28	KLEIN EXITO	Spring
29	Petirrojo	Spring
30	VALKI 141-R-E-34	Spring
31	KM 780-92	Spring
32	Ballobar	Spring
33	Aus27813	Spring
34	Aus27429	Spring
35	Aus28050	Spring
36	Aus27454	Spring
